# Synthesis and Characterization of Nanocrystalline Boron-Nitride Thin Films by Ion Milling and Thermal Treatment for Tribological Coatings: An Approach to Quantifying the Growth Dynamic Process

**DOI:** 10.3390/ma15051761

**Published:** 2022-02-25

**Authors:** Carlos Alberto Monezi, Korneli Grigoriev Grigorov, Aleksandar Tsanev, Armstrong Godoy, Antonio Augusto Couto, Arnaldo Oliveira Lima, Georgi Avdeev, Roumen Iankov, Marcos Massi

**Affiliations:** 1 School of Engineering—PPGEMN, Mackenzie Presbyterian University, Rua da Consolação, 930, São Paulo 01302-907, SP, Brazil; carlos.oliveira@mackenzie.br (C.A.M.); antonioaugusto.couto@mackenzie.br (A.A.C.); 2 Space Research and Technology Institute, Bulgarian Academy of Sciences, Acad. G. Bonchev Str. Bl. 1, 1113 Sofia, Bulgaria; 3 Institute of General and Inorganic Chemistry, Bulgarian Academy of Sciences, Acad. G. Bonchev Str., Bl. 11, 1113 Sofia, Bulgaria; tsanew@abv.bg; 4 Plasmas and Processes Laboratory—LPP, Technological Institute of Aeronautics—ITA, Praça Mal. Eduardo Gomes, 50, São José dos Campos 12228-900, SP, Brazil; godoyajr@gmail.com; 5 Surface Phenomena Laboratory, Escola Politécnica da Universidade de São Paulo, São Paulo 05508-010, SP, Brazil; arnaldo_olima@usp.br; 6 Rostislaw Kaischew Institute of Physical Chemistry, Bulgarian Academy of Sciences, Acad. G. Bonchev Str., Bl. 11, 1113 Sofia, Bulgaria; g_avdeev@abv.bg; 7 Institute of Mechanics, Bulgarian Academy of Sciences, Acad. G. Bontchev St., Bl. 4, 1113 Sofia, Bulgaria; iankovr@yahoo.com

**Keywords:** energy-dependent growth constants, tribology, plasma-enhanced surface modification, hardness improvement, reactive ion etching (RIE), nanocrystalline BN

## Abstract

Hexagonal boron-nitride nanoparticle coating was deposited on AISI 1045 steel surface. The deposition process included a transformation of B-containing thin organic film into nanocrystalline BN using two methods: thermal annealing at 450–850 °C and reactive ion etching in Ar/N_2_ plasma. The film structure, phases, and film morphology of deposited nanoparticles of boron nitride on AISI 1045 steel were characterized by XPS, XRD, and EDS. Post-annealing at 450 °C does not lead to the formation of a BN phase in the layer. A non-stoichiometric BN phase with nitrogen deficiency appears at 650 °C. At 850 °C annealing, the formed BN phase is completely stoichiometric. The effects of deposited and incorporated BN on the friction and hardness properties of AISI 1045 steel were also studied. The post-annealing process improved the hardness from 5.35 to 11.4 GPa, showing a pronounced linear temperature dependence. An original approach was adopted to quantify the energy-dependent growth constants based on the indentation load-discharge curves measured on samples treated under different conditions. Those constants describe the rate of the reactions and the type of interdiffusion process characteristic for each material used. This approach can partially fulfill the role of the Rutherford backscattering spectrometry profile, which is an expensive and time-consuming process, mainly when light elements such as boron and nitrogen are used.

## 1. Introduction

Solid lubricants, such as diamond-like carbon (DLC) and MoS_2_ coatings, present a wide range of favorable tribological behavior, as their friction coefficients suggest that they can help reduce the friction and wear on mechanical parts, such as in an engine, and significantly save costs. The wear properties of the DLC coatings as a boundary lubricant depend on the hydrogen content [1]. Other complex compounds, including TiSiN_x_ or Ti-6Al-4V, have excellent corrosion resistance and good strength [2], but they present much higher wear. An attractive alternative as a functional coating [3] that provides mechanical protection is h-BN, which, combined with cubic-BN (c-BN) in a multilayer configuration, exhibits high hardness with a low friction coefficient. Boron nitride is a carbon isoelectronic material with two phases: hexagonal and cubic, which is similar to a diamond-like phase. Other advantages of this allotropic material include its remarkable thermal stability, as well as its mechanical, optical, and electrical properties, such as hardness, chemical inertness, lubricity, and high thermal conductivity. Additionally, h-BN films present deep-UV luminescence and, consequently, unique field-emission properties that serve a variety of advanced applications [4,5]. However, most of the unique properties of this material depend on the particle shape and crystalline size, a phenomenon extensively described in [6]. Powdered h-BN can significantly reduce friction and wear when mixed at low concentrations (as little as ∼1 wt.%) with liquid lubricants (oils, greasers, and fuels). Fundamental investigations of the properties of the h-BN lubricant found that h-BN is generally less effective than other solid lubricants, except for in high-temperature applications. However, a series of sliding experiments showed some curious behavior when added to lubricating oil, e.g., in the case of sliding of bearing steel, BN slightly increased the coefficient of friction but drastically decreased wear [6]. With tribological properties similar to those of graphite, the hexagonal phase is applied as a solid lubricant [7] whereas, similar to diamond, the cubic phase has high hardness (the second hardest material in nature) and a wide band gap. h-BN has a structure, similar to that of graphite, in which the B and N atoms are bonded alternately in hexagonal rings in the plane, forming two-dimensional sheets, which are held together by van der Waals forces but are electronically insulated with an indirect gap of ∼5.95 eV [8]. The h-BN crystals offer remarkable mechanical strength [9], chemical stability [10], and thermal conductivity [11] due to their covalent bonds in the basal planes. This soft material with a low friction coefficient (FC) is a lubricant at high and low temperatures [12], an electrical insulator, and thermally conductive, plus has very good corrosion-resistance properties. It has wide applications as a solid lubricant at high temperatures in any environment [13] and is applied extensively in high temperature, high humidity metal-forming dies and other metalworking processes. The c-BN phase has up to 70 GPa greater hardness than the h-BN phase. Furthermore, due to its high chemical and thermal stability, c-BN is much better than diamond as a tool for ferrous products [14]. The properties of c-BN films are similar to those of diamond, but c-BN differs from diamond in terms of its high oxidation temperature (~1200 °C), thermal stability (~1500 °C), and chemical stability for ferrous materials (c-BN is inert to iron, and diamond is not). These superior properties relative to diamond allow for its use in tribological applications [15]. Thus, c-BN is undoubtedly an excellent material for hard coatings in industrial and scientific applications [16,17,18,19]. However, c-BN shows notable surface-compressive stresses. Differences in the surface tension between the substrate and the deposited film often result in weak adhesion and subsequent peeling of the layer. As previously mentioned, these stresses due to lattice mismatch have been successfully reduced by post-annealing and sequential multi-film growth [20,21,22,23].

To grow c-BN thin films, several deposition techniques have been successfully used, including chemical vapor deposition (CVD) and physical vapor deposition (PVD). Radio-frequency (RF) magnetron sputtering is a PVD method that is commonly employed to deposit c-BN films. Hexagonal h-BN is used as a target material in most cases for BN deposition by RF magnetron sputtering. Most PVD techniques rely on electron assistance with a positively polarized substrate or energetic ion bombardment with a negatively biased substrate. However, one crucial problem that must be solved is that the energetic ions induce defects in the growing film, which could be the origin of high stresses up to 10 GPa. This could result in weak adhesion of the coatings. The c-BN formation model developed by McKenzie [24] assumes that ion bombardment creates compressive stresses, and the formation of c-BN is possible only when the constraints exceed a certain critical value. Robertson [25] developed a theory that the formation of sp^3^-like bonds are established by a process called sub-implantation. These growth processes are complex, which motivated us to consider an original experiment to transform bond sp^2^ into sp^3^ because of ion implantation. Theoretical calculations to establish the energy, dose, and nature of the ions performed by Deyneka et al. [26] indicate significantly increased grain sizes of c-BN and overall crystallinity improvement when using high substrate temperatures (i.e., T_S_ > 800 °C) during c-BN growth, presumably due to increased atomic diffusivity.

In the present study, we report the results of our attempts to obtain a nanocrystalline BN coating on AISI 1045 steel. For this purpose, a boron-rich organic paste was deposited onto the steel surface through ion-milling RIE plasma. Using post-annealing, this coverage was transformed into a nanocrystalline boron-nitride thin film. Characterization with different spectral methods during the formation of the BN layer found that the effect of post-annealing not only improved layer stoichiometry but also improved the steel hardness and wear. Compared to previous literature studies discussed above, we offer a relatively inexpensive method to obtain the nanocrystalline coating. In addition, the approach proposed in the article for determining the basic physical parameters of the layer-formation process and its quality also has the advantage of being inexpensive.

## 2. Materials and Methods

### 2.1. Sample Preparation and Deposition

Ten mirror-polished AISI 1045 (steel manufactured by Gerdau, Barão de Cocais, Minas Gerais, Brazil) samples were covered with a slightly viscous, homogeneous, B-containing paste. Half were subjected to thermal annealing at 450–850 °C in running N_2_-gas for 1 h. The other half underwent reactive ion etching (RIE) with high-density N_2_/Ar-mixture plasma for 10 min at different power levels (100–300 W).

The paste containing B consisted of 3 g of pure (99.99%) element, which was ground for 20 min in agate chemical mortar, and 1.4 g of PEG 20000 (Xilong, Shantou, China) surfactant was subsequently added in portions for the next 20 min. Then, 450 μL of acetylacetone was added and ground again. Finally, one drop (15 μL) of triton X-100 was added to achieve regular and homogenous content.

The annealed samples were called A1, A2, and A3 (450, 650, and 850 °C), whereas the plasma-treated samples were named A(100), A(200), and A(300) (270, 320, and 380 °C). The samples that followed the thermal procedure underwent one hour in a quartz tube placed in a programmable oven at 450 °C, 650 °C, and 850 °C in running N_2_.

**Figure 1:**
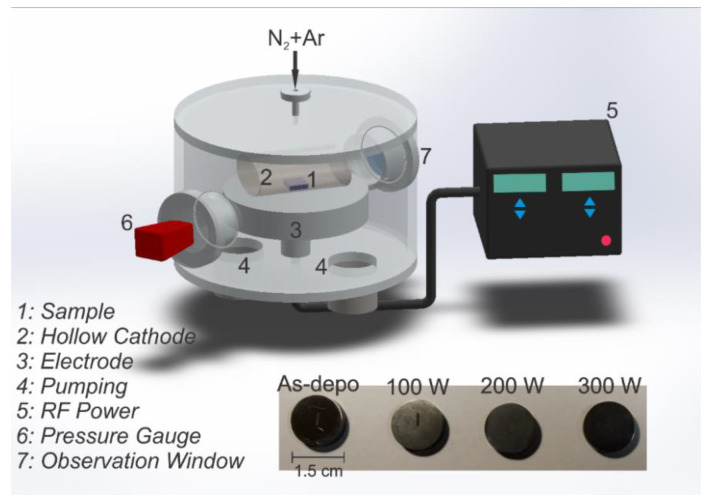
Schematic diagram of the reactor and photographs of the samples as deposited and treated for 10 min in an N_2_ atmosphere under powers of 100 W, 200 W, and 300 W.

The plasma reactor was equipped with a hollow cathode and a capacitive plasma source using a 13.56 MHz radio frequency (RF) ENI model ACG-10B-01 (Andover, MA USA) as a power source [26]. The hollow cathode made of high purity (99.99%) titanium was placed directly on the reactor electrode, as shown in Figure 1. Each sample was subjected to plasma treatment for 600 s with RF power of 100 W, 200 W, and 300 W, which caused negative bias of 210 V, 310 V, and 380 V, respectively. The working and residual pressures were 13.3 Pa and 5.0 × 10^−2^ Pa, respectively. The vacuum regime was achieved with the aid of a root and a mechanical pump. The chemical and physical phenomena that occur inside a hollow cathode are much more intense than in a conventional configuration [27], increasing the adatom surface mobility to a greater degree, which leads to more efficient incorporation of nitrogen into the samples. The heavier Ar gas promotes surface defects, allowing better adhesion of BN phases. Primary and secondary emitted electrons multi-reflected from the hollow cathode surface can suffer several collisions with the gas along their paths, enhancing ionization and, consequently, the plasma density [28,29].

At the end of the treatments, both the annealed and RIE-treated samples were polished with 200 nm grain size diamond-abrasive paste to remove the excess residues.

### 2.2. Hardness Measurements

The hardness of the samples was measured using Bruker’s Triboindenter Hysitrons Ti-950 (Billerica, MA, USA), with a diamond tip and Berkovich geometry. A load of 1 mN was used with a time of 5 s for loading, 2 s for landing, and 5 s for unloading. In each sample, the hardness was measured at between 5 and 7 different points, and the mean and standard deviation were calculated.

### 2.3. XRD Analysis

X-ray diffraction spectra (XRD) of all samples were measured in Theta-2Theta configuration with step size [°2Th] = 0.02 and scan step = 1 [s] with a Philips PW1050 diffractometer from 10 to 90° using a Cu-cathode (λ = 1.54060 [Å]).

### 2.4. Coefficient of Friction (FC) Measurements

The FC was measured using a Tribometer UMT-2 by Bruker Inc. (Billerica, MA, USA), with an AISI 52100 steel ball with a radius of 5 mm as a counter body. The tests were conducted on a dry surface and included two parts. The first sequence consisted of a reciprocating linear movement of 4 mm length with a load ramp from 0 to 10 N measured for 1 h. The second implied a constant normal load of 10 N for 4 h. Thus, FC assumes the tangential force to the normal force given by the equipment.

### 2.5. XPS Analysis

Five X-ray photoelectron-spectroscopy (XPS) studies were performed on a VG Escalab II system, using Al Kα radiation with an energy of 1486.6 eV. The chamber pressure was 1 × 10^−9^ Pa. The C1s line of adventitious carbon at 284.6 eV was used as internal standard to calibrate the binding energies. The photoelectron spectra were corrected by subtracting a Shirley-type background and were quantified using the peak area and Scofield’s photo-ionization cross section. The accuracy of the binding energy (BE) measured was ±0.2 eV.

### 2.6. EDS/SEM Measurements

Scanning electron microscopy (SEM) analysis was used in conjunction with energy-dispersive X-ray spectroscopy (EDS), which is a chemical microanalysis technique that detects X-rays emitted from the elemental composition of the sample during electron-beam bombardment to characterize the elemental composition of the analyzed volume.

## 3. Results and Discussion

### 3.1. Hardness Measurements

**Figure 2:**
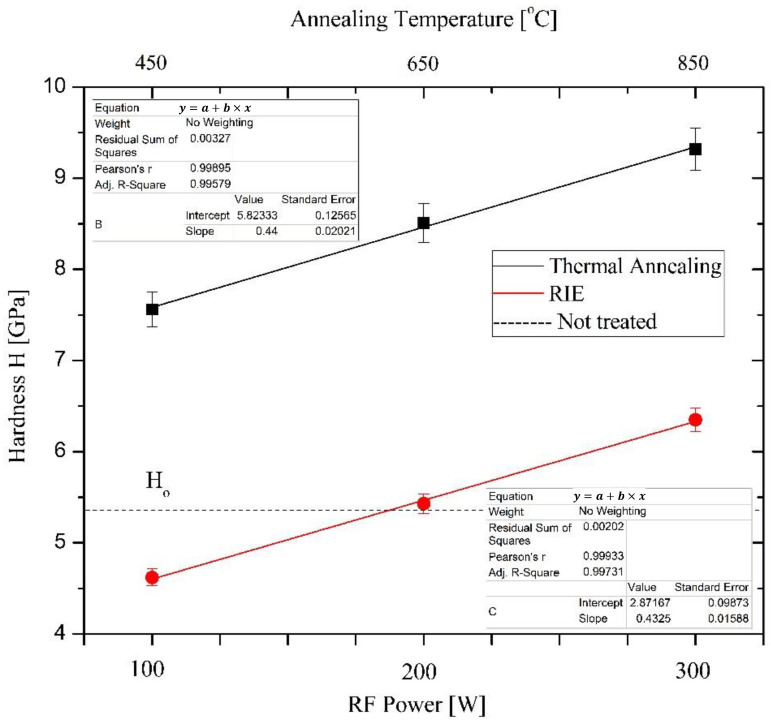
Hardness plots of the annealed samples in an N_2_ atmosphere at different temperatures and for RIE process treated in an Ar-N_2_ gas mixture for 10 min by different RF power. Round circles—RIE-treated; squares—temperature-treated.

As discussed previously, both treated batches were subjected to hardness measurements performed with a maximum load force of 1 mN. Additional details related to the technical approach are discussed in Section 3.6. Figure 2 presents both hardness measurements. Note that the temperature range on the upper scale refers only to annealed samples. The RIE process was independent, and for each power, different temperatures were measured using a pyrometer. As shown in Figure 2, both hardness dependences are linear and parallel to each other. This suggests that the mechanisms governing the formation of BN phases are temperature dependent. Each point represents the mean value taken from five to seven measurements expressed in histograms (not presented). The temperature-treated samples have a maximum standard deviation of 23%, whereas the RIE-treated samples have an 8% maximum standard deviation. The value of the reference sample (H_o_) is marked with a dashed line. Growth mechanisms and processes of phase formation, morphology, elemental composition, grain size, and friction coefficients are also revealed.

### 3.2. XRD Analysis

**Figure 3:**
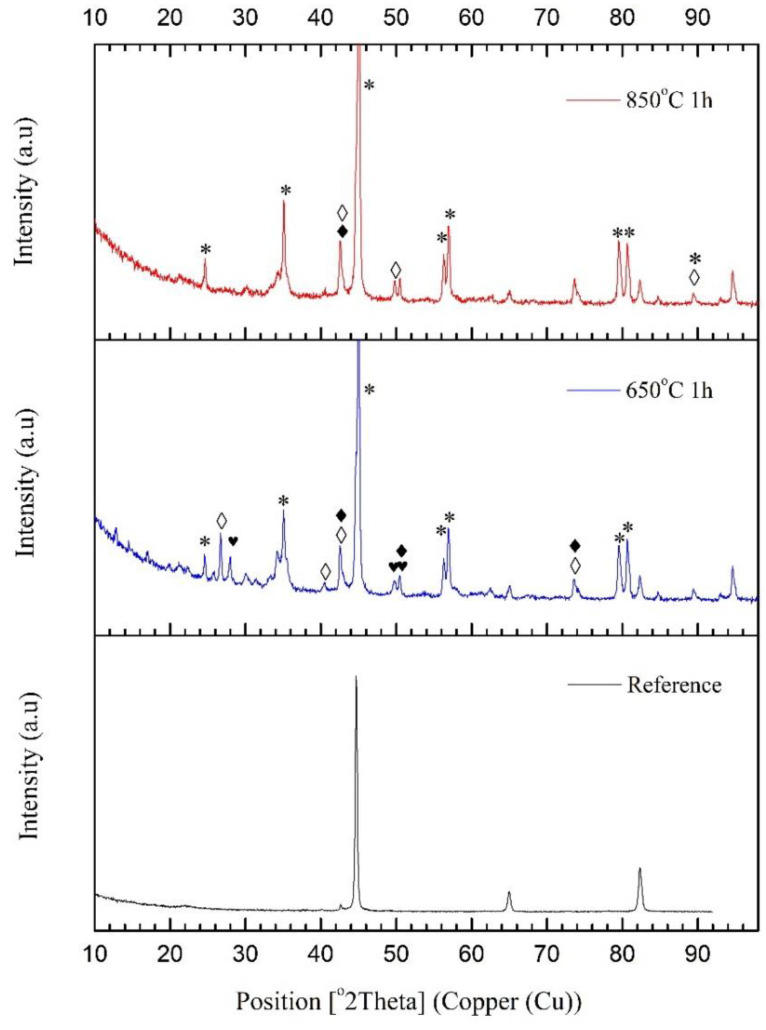
XRD patterns of the reference sample, together with the samples treated for 1 h in an N_2_ atmosphere at different temperatures. ◊—nanostructured h-BN phase; ♦—a cubic (c-BN) phase; 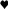
—iron carbide (C_3_Fe_7_); *—iron-boride phase, Fe_2_B.

**Figure 4:**
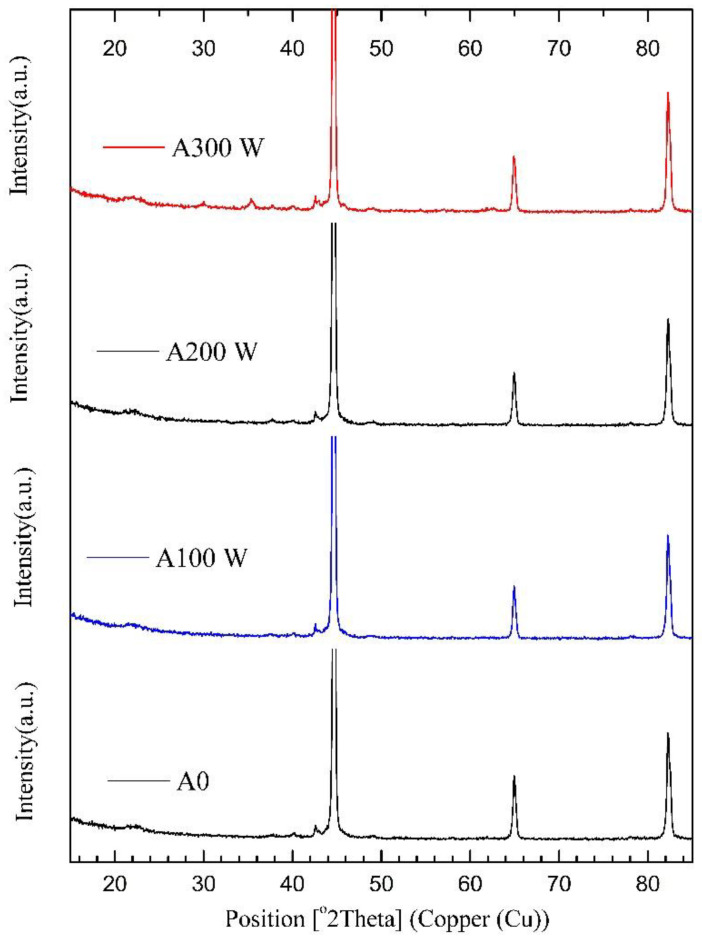
XRD patterns of RIE-treated samples—10 min at different RF power in 13.3 Pa (100 mTorr) N_2_-Ar gas mixture. All pronounced peaks belong to cubic iron, and the minor peaks belong to cubic Fe_3_O_4_.

Figure 3 and Figure 4 present the X-ray diffraction (XRD) spectra of all samples from both methods. The reference spectrum consists of cohenite (Fe, Ni, Co) 3 C up to 3% and Iron (the most pronounced peaks), with a mean hardness value of 5.39 GPa (Mohs scale) and a relatively high FC = 0.52. The temperature treatment of boron-doped samples at 650 °C (see Figure 3) yielded a rich spectrum consisting of nanostructured h-BN (◊) (reference code 98-016-8892) with a mean grain size of 41 nm deduced by reflections from plan (002) using the Scherer formula. A c-BN phase (♦) is detectable (reference code 00-015-05000); however, the mean grain size could not be estimated, as thickening of the spectral lines occurred because of peak superposition. The concentrations of h-BN and c-BN are 53% and 47%, excluding the other constituents. The other phases are iron boride (*) Fe_2_B (reference code 98-016-0791) (83%) and Iron Carbide C_3_Fe_7_ (17%), excluding the other constituents. Therefore, B-based phases have a 36% concentration, and Fe-based phases have a 64% concentration.

The samples treated at 850 °C show a c-BN phase (reference code 00-015-0500) and an h-BN phase (ref code 00-026-0773) with a mean grain size of 34 nm, as well as iron-boride (*) Fe_2_B (ref. code 98-016-0791). The remaining reflections are from iron boride. The boron phases are 19%, of which 10% is c-BN and 9% h-BN.

X-ray radiation is very energetic, and the reflected signal depends mainly on the radiation length (cathode type—Cu, Cr, Co, W, etc.), as well as on the nature of the characterized material—nitrides, carbides, oxides, or intermetallic compounds—and not on the grain size of the matrix. If a copper cathode is used, the depth could reach up to 5 μm, but it is often at the scale of 1 to 2 μm. According to the (H-h) displacement graphs (see Section 3.6. Growth Dynamics of the BN Phases ), the treated zone of the samples reached 70 nm. Thus, the method takes an integral signal from a certain depth, and the thin surface composition has less of an impact in the case of theta-2theta geometry. As discussed in the nanoindentation section, the modified thickness reached 500 nm, which provides a reasonable accuracy for this method. This method reveals that films treated at 650 °C for 1 h in an N_2_ atmosphere consist of 36% boron-based phases, 17% iron carbide, and 47% iron boride, while the samples treated at 850 °C consist of 19% boron-based phases and 81% iron boride. The main peak <111> of the c-BN phase of the sample treated at 650 °C (reference code 00-015-0500), which usually appears at 43.254° (*a* = *b* = *c* = 3.62 Å) with spacing of *d* = 2.09 Å, is shifted to 42.61^o^. Applying the Bragg rule for Cu Kα radiation yields interplanar spacing of *d* = 2.12 Å, corresponding to *a* = 3.67 Å, keeping in mind that $\frac{1}{d}=\frac{h^{2}+k^{2}+l^{2}}{a^{2}}$ for the cubic symmetry. It appears that the cell parameter is greater with 0.05 Å, suggesting a deficit of N_2_ atoms forming the cell. However, the same is not applicable for the h-BN phases that do not suffer any lack of nitrogen atoms. Following the same procedure for the c-BN phase (reference code 00-015-0500) for the samples annealed at 650 °C, we found *a* = 3.65 Å, which is closer to the original state, 3.62 Å.

The RIE-treated samples present different spectra (Figure 4) than those of the temperature-treated samples. The most pronounced peaks at the higher power (300 W) spectrum at 44.6°, 65.03°, and 82.43° belong to the cubic Fe (03-065-4899), and all the other (minor) peaks belong to a cubic Fe_3_O_4_ (01-089-2355) iron oxide. Apparently, B-containing phases are not detectable via this method (BN phase was confirmed by XPS measurements only for the A300 sample). Figure 4 also shows that the Fe peak (211) for both 200 W and 300 W samples suffers a notable intensity increase—from 1480 to 1730 a.u.—increasing the grain size from 20 nm to 35 nm, respectively. The same trend is evident for Fe (200) planes, where the intensity ranges from 736 to 790 a.u., and the grain sizes range from 20.3 to 27 nm. The latter results in a 55% increase in grain size, which produces better organized and aligned grains in a less densely packed structure. The latter opens a larger possibility of interface diffusion. It is important to remember that the iron samples have simple cubic packing wherein the free space is proportional to the r^2^ of the spheres.

### 3.3. EDS/SEM Measurements

Scanning electron microscopy (SEM) analysis was used in conjunction with energy-dispersive X-ray spectroscopy (EDS), which is a chemical microanalysis technique that detects x-rays emitted from the elemental composition of the sample during electron-beam bombardment to characterize the elemental composition of the analyzed volume. Quantitative results are available using standards. We present elemental maps for both treated samples with different mathematical filters applied, such as backscattered-electron-composition (BEC) images to distinguish heavier elements from lighter elements; secondary-electron images (SEI), where morphology appears clearly; and a serial peripheral interface (SPI), which both detectors perform at the same time, with one half observing back-reflected electrons and the other observing secondary electrons.

**Figure 5:**
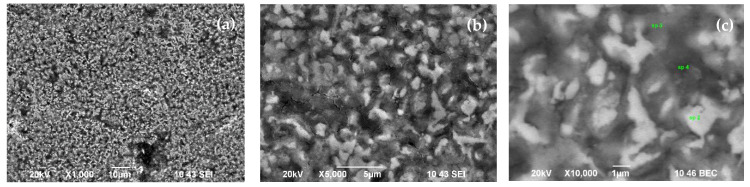
Secondary electron images (SEI) (**a**,**b**) and backscattered electron composition (BEC) (**c**) of the sample treated at 650 °C.

**Figure 6:**
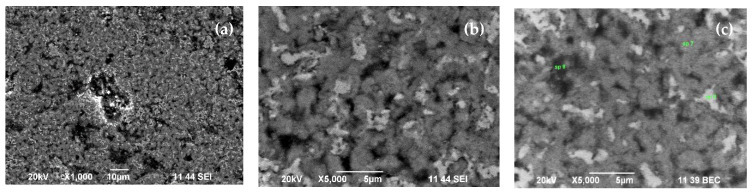
Secondary electron images (SEI) (**a**,**b**) and backscattered electron composition (BEC) (**c**) of the sample treated at 850 °C.

As the applied filters help to distinguish heavier elements from lighter elements, Figure 5b and Figure 6b show where the lighter spots (associated with lighter BN) have a higher concentration in the same studied area. The results of EDS semiquantitative elemental composition provided in Table 1 also coincidence with the XRD analyses, where the boron phases at 650 °C/850 °C are found 39/19 (at. %). In general, both sets of SEM images display a smooth, granular surface without major cracks where elements have a homogenous distribution. In the elemental mapping summarized in Table 1, carbon and the nitrogen are missing, as they were not detectable, even though they are part of the composition, such as borides and carbides Fe_2_B and C_3_Fe_7_.

### 3.4. XPS Analysis

Compared to other experimental methods used in the present study, XPS is sensitive to the uppermost atomic monolayers. Its depth of analysis (around 4–6 nm) is defined as three times the mean free path of the photoemitted electrons in the solid, depending on their kinetic energy.

**Figure 7:**
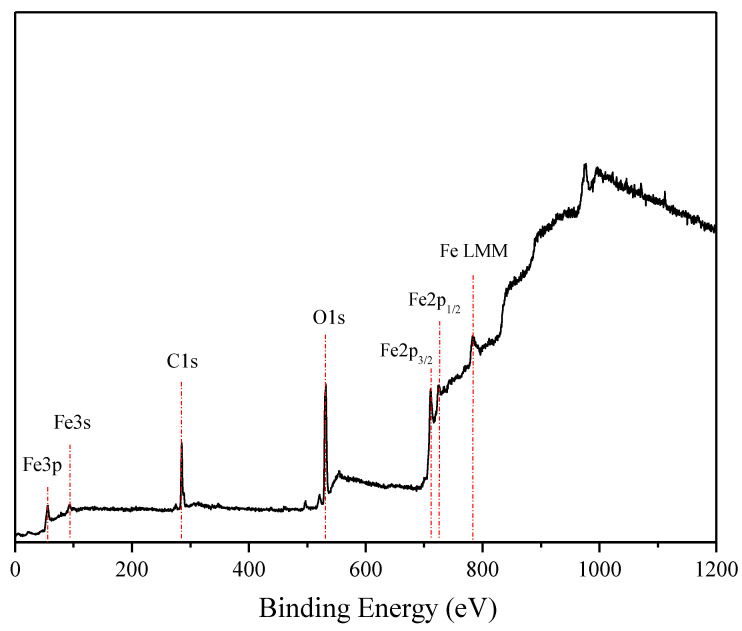
Wide XPS scan of AISI 1045 carbon-steel polished referent samples.

Figure 7 presents a wide spectrum of polished AISI 1045 reference samples. Besides the Fe_2p_ peaks and Fe LMM structure due to Auger transitions, only C1s- and O1s- peaks are visible. Note that about 60% of the XPS signal comes from the uppermost atomic surface layer and attenuates exponentially at depths below the surface. Therefore, the latter peaks can be attributed to carbon- and oxygen-containing adsorbed particles.

XPS analysis of the annealed samples aimed to establish the quantitative and qualitative composition of the sample surface, as well as to identify the products of precursor conversion due to past chemical reactions. Table 2 summarizes the elemental concentrations for the annealed samples according to the XPS analysis. The above-mentioned higher sensitivity of XPS to the uppermost surface atomic layer can explain the relatively higher concentrations of carbon and oxygen attributed to adsorbed species, which, to some extent, shield the signal from the B-containing film below.

Sample 1 (450 °C; see 1/450 °C in Table 2) shows that the boron concentration is the lowest, at 9 at%, which indicates that the rate of chemical conversion is insignificant at this temperature. Here, the BN phase is below 10%. The proof of this is the small concentration of nitrogen, which is less than 2 at%. Here, the layer is thin. The concentration of Fe that is a constituent element of the pad is higher, at 4.2 at%, suggesting that the overlying B-containing layer is thin. For the 2/650 °C and 3/850 °C samples (see Table 2), the higher temperatures decrease the percentage of oxygen and increase that of carbon, while the concentration of the remaining elements—iron and sodium—remains constant. The treatments at higher temperatures lead to a higher degree of oxygen combustion, which leads mainly to increased carbon content. The latter ends with the film surface masked with carbon and hydrocarbon combustion products, leading to further agglomeration of the particles. At the higher temperature, the interaction between the boron precursor and the nitrogen flow is activated, resulting in more nanocrystalline boron nitride.

**Figure 8:**
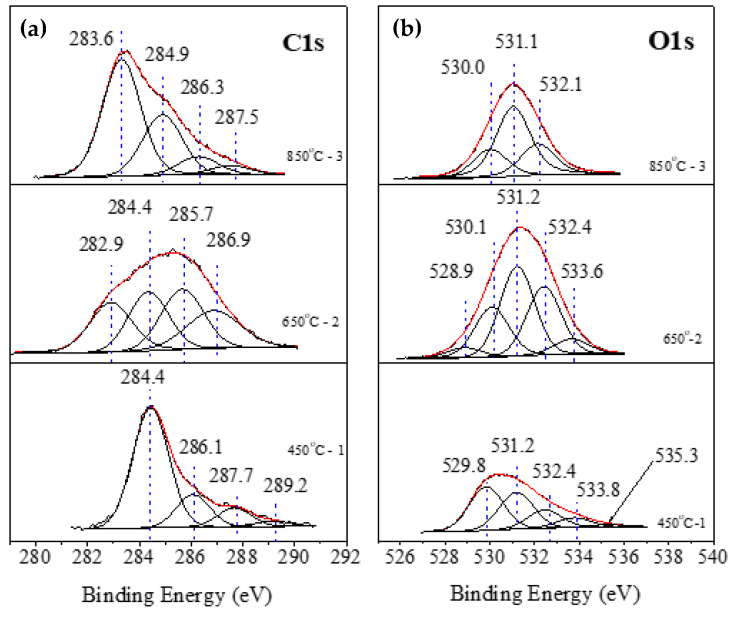
(**a**,**b**). XPS high-resolution carbon 1s and oxygen 1s core-level spectra.

Characterizing the chemical composition of the surface layers of the samples requires a detailed study of the shape of the various photoelectron peaks. Figure 8a presents the C1s peaks for all samples. They are very broad because there are many different C-containing chemical bonds. All the spectra can be deconvoluted into four peaks. The first group of peak contributions, located in the range of 282.9–283.3 eV, is characteristic of metal carbides [30,31]. The second group of contributions at 284.4–284.9 eV could be associated with C-C bonds [32]. The peak contributions at 285.7 eV, 286.3, and 286.9-287.5 eV could be attributed to C-H, C-O-C, and C=O bonds, respectively [33]. Sample 1 (450 °C) exhibits an additional C 1s peak at 289.1 eV, associated with COOH groups [34,35].

Furthermore, the O1s photoelectron regions can be fitted with several peak contributions (Figure 8b). The first group of peaks at about 530 eV can be associated with O in the frame of FeO [36]. The second peak-contribution group at about 531.2 eV can be attributed to the presence of C = O or –OH¯ groups [37]. The third group of peaks at about 532.4 eV is due to the presence of crystal-hydrated water in the composition of Na_2_B_4_O_7_ · 10H_2_O [38]. The last peak, found only in sample 2, can be assigned to O bonded with B and Na in the compound Na_2_B_4_O_7_ · 10H_2_O [37]. Again, in sample 2, another peak at 528.9 eV can be associated with some amount of partially reduced non-stoichiometric iron oxide (Fe_2_O_x_).

**Figure 9:**
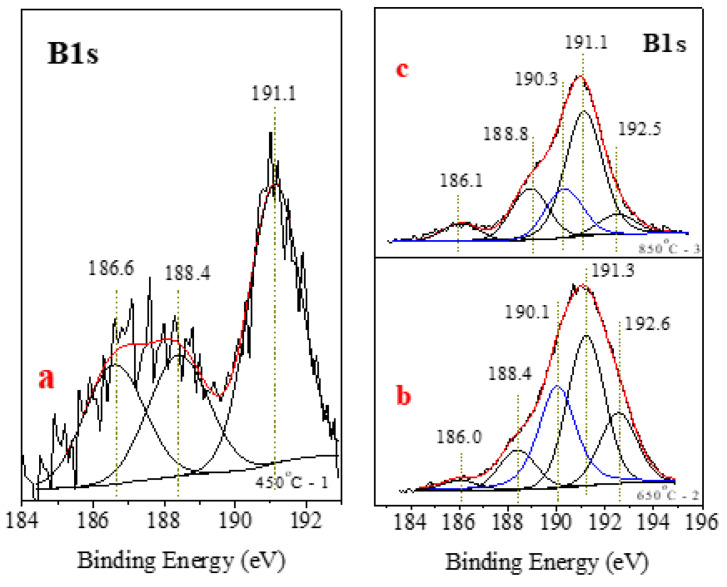
(**a**–**c**). XPS spectra of the B1s core level for the annealed samples.

Figure 9a,b displays the XPS spectra of the B1s core level for the annealed samples. The final annealing temperatures are indicated in the figure. The spectrum corresponding to 450 °C annealing is broad and can be fitted with three peak contributions. None of them can be associated with BN phases. The first and second peak contributions at 186.6 and 188.4 eV are associated with B–B bonds of elemental boron [39], BC, and Fe–B phases (Fe_2_B, FeB) [40]. The third peak at 191.1 eV is associated with the organic phases of B [40].

The spectra of samples 2 and 3 (Figure 9b,c) deserve more attention. The spectra can be deconvoluted into five peaks. The first and second photoelectron lines located at 186.1 and in the range of 188.4–188.8 eV are assigned to formed phases of B_x_C, Fe_2_B, and FeB. The fourth and fifth peaks are associated with the organic phases of B and B in Na_2_B_4_O_7_ · 10H_2_O [39,40,41]. The third peak at 190.1–190.3 eV (samples 2 and 3) belongs to the formed phase of BN [42,43]. This peak of sample 2 is more intense than that of sample 3, and its integrated area is approximately 31% of the area of the whole spectrum. This value is close to the calculated value from XRD analysis. Here, the value is lower due to the screening effect of the surface carbon.

For sample 3, the area of the corresponding BN phase at 190.3 eV is 18% of the area of the whole spectrum. Table 3 summarizes the percentages of the boron phases.

**Figure 10:**
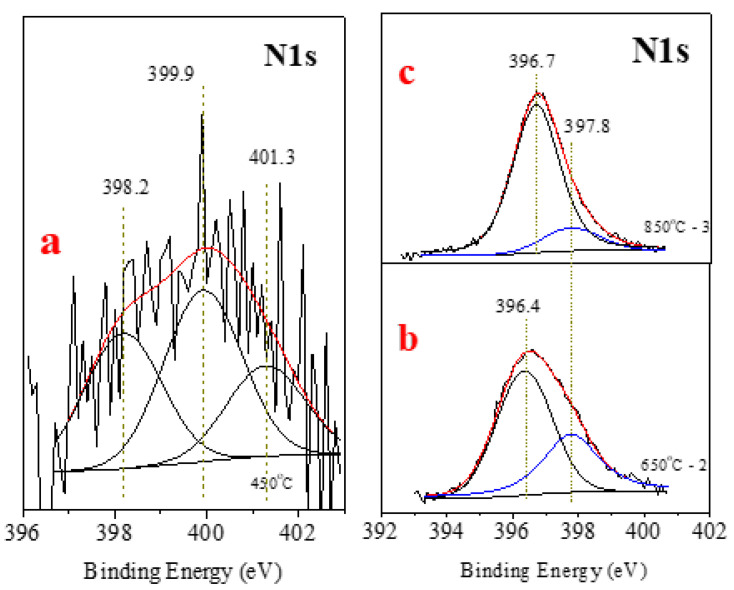
(**a**–**c**). XPS spectra of the N1s core level for the annealed samples.

For sample 1, the N1s XPS spectrum is provided in Figure 10a, and no peaks are associated with the BN phase. Three peak contributions correspond to N bonded with/in organic compounds, such as the imine groups (R = N − R groups (398.2 eV)) [44], amine groups (C – NR2 groups (399.9 eV)) [45], or alkyl ammonium groups (NR4+ (401.3 eV)) [46]. Figure 10b,c shows N1s photoelectron spectra for samples 2 and 3. The peak position for the BN phase is 397.8 eV. Consistent with the data reported above, the peak intensity in sample 3 is lower than that in sample 2. The peaks at 396.4–396.6 eV are associated with N in the composition of FeN [47].

The reported XPS results show that the BN phase is formed in annealed samples 2 and 3. The precise calculation of its stoichiometry can be done after area normalizations (to their ionization cross sections) of B 1s and N 1s peaks corresponding to the BN phase. For sample 2, these peaks are located at 190.1 eV and 396.4 eV. The ratio of their normalized areas gives a B:N concentration ratio close to 2. The shortage of nitrogen resulting from non-stoichiometric BN phase formation is also seen in the thinner XRD (111) peak shift towards the smaller theta-2theta. A significantly better result was obtained for sample 3, considering the B 1s and N 1s peaks of BN at 190.1 eV and 396.7 eV, respectively. The concentration ratio is 1.1, corresponding to a perfect BN stoichiometry. In conclusion, sample 1, annealed at 450 °C, is composed of a mixture of organic B. The temperature was insufficient to form a BN phase. For samples two and three, annealed at 650 °C and 850 °C, respectively, a BN phase was formed. According to the analyses, the sample annealed at a lower temperature shows a slightly higher BN phase concentration within the instrumental error (~1 at%), but to some extent, it is non-stoichiometric. A stoichiometric BN phase formed after annealing at 850 °C.

### 3.5. Friction Coefficient (FC) Measurements

**Figure 11:**
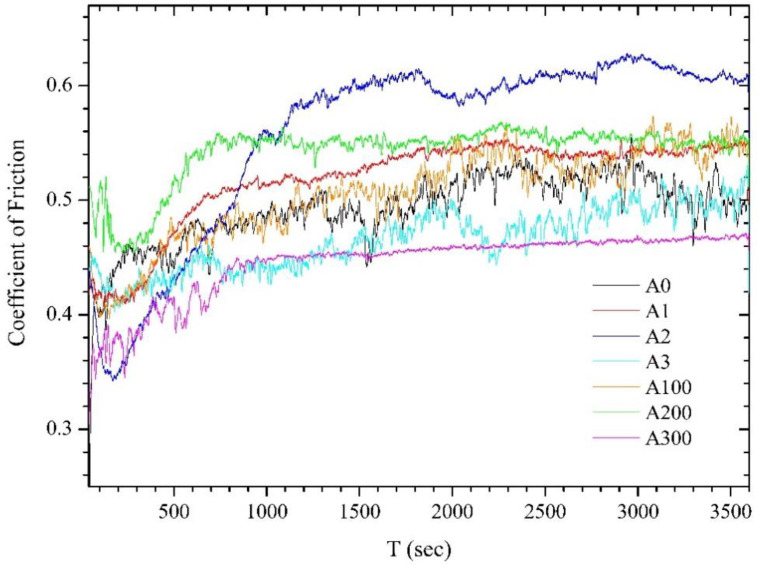
FC curves for the annealed samples (A1–A3) and RIE samples (A100–A300) at increasing normal load from 0 to 10 N for 1 h. A0 refers to the referent sample.

A linear reciprocating test was performed on a dry surface and using a ramp load from 0 to 10 N for 1 h. The obtained results are presented in Figure 11. Interestingly, the FC curves are situated on both sides of the reference-sample result, independently, in which the treatment was used. Sample A2 (Figure 10, blue line), annealed at 650 °C, shows the highest FC, at 0.6, and according to the XRD measurements, the B-based phases (c-BN + h-BN) have a 36% concentration, whereas the Fe-based phases have a 64% concentration (iron carbide and iron boride). The FC at lower loads starts at 0.34 and gradually increases. The higher FC value could be explained by significant amounts of C_3_Fe_7_ and F_2_B. The extended ramp up to 1000 s could be associated with the poor adhesion of nanocrystalline h-BN grains in the matrix due to insufficiently high temperature.

In contrast, A3 (850 °C) presents an FC of 0.47—almost 30% less than that of sample A2. The AISI 1045 average FC under 50 N load is about 0.75 [6]. The small variations in FC values as a function of time probably refer to transfer of h-BN to the proofing ball and its oxidation; however, this is just a hypothesis, as the proofing balls were not analyzed. RIE treatment resulted in both higher and lower FE values: 0.54 for A200 W and 0.45 for A300 W, which is the lowest obtained value. The FCs of A200 W and A300 W remain stable in the long term. As suggested previously, the intense ion bombardment created surface defects with dislocations, vacancies, and increased porosity, serving as nucleation centers for the formation of BN phases, as their presence remained intact after subsequent polishing of the samples. Even created by different techniques, A3 and A300W have the lowest FCs—0.47 and 0.45—and hardness of 9.4 GPa and 6.35 Gpa, respectively.

### 3.6. Growth Dynamics of the BN Phases

During the annealing and RIE treatment of the samples, interdiffusion phenomena occurred between both the substrate and film media, leading to extensive phase formation. Temperatures, in general, play an extremely important role in solid kinetic processes and define temperature-affected variables, such as diffusivity and the rate of the chemical reaction plotted logarithmically versus 1/T (K). During these treatments, surface or bulk-diffusion processes controlled by transport via grain boundaries, cracks, or vacancies are common. Bulk diffusion occurs at very high temperatures and is characteristic of single-atom paths. Processes controlled by transport via grain boundaries, cracks, or vacancies depend on the grain size and film density. Many works [48,49,50] have studied the effect of grain size and grain boundaries on the mechanical properties of materials. Different crystallite sizes of similar materials result in differing hardness. Huang et al. studied the effect of crystallite size on hardness in AlCrNbSiTiV [51] and found that grains of 50.6 nm and 15.5 nm result in a differing hardness of 7.5 GPA. Vacancies act as transition centers that promote material transport, and according to the gradient model of vacancy concentration [52], their concentration is highest near the surface and decreases within the sample, predicting a depth-dependent diffusion coefficient. However, dynamic manner, reaction rate, and diffusion constants depend on the temperature range and the complex nature of the acting film constituents.

**Figure 12:**
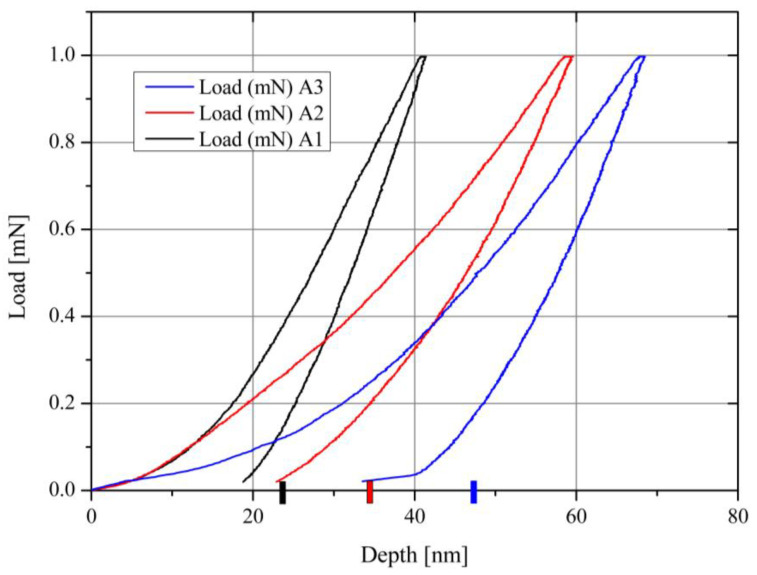
Charge–discharge indentation curves for samples A1, A2, and A3 (Table 1) at a maximum load of 1 mN. Color markers identify the contact depth hc of the indenter at maximum loads.

The load-discharge indentation curves illustrated in Figure 12 were acquired for the treated samples at different temperatures, resulting in different shapes depending on the sample hardness if measured at identical loads. Gradually increasing the load force at certain depths of penetration decreases the hardness. According to the Oliver–Pharr method [53], the adopted evaluation parameters are *h_c_* = *h_m_* − *ε*(*h_m_* − *h_r_*) and *h_e_* = *ε*(*h_m_* − *h_r_*). Here, *ε* is the form factor (0.7268 for Berkovich-type indenter); *h_e_, h_m_,* and *h_r_* are the elastic, maximum, and residual depths, respectively; whereas *h_c_* is the contact depth of the indenter with the material at maximum load. The hardness is estimated as *H = F_max_/A_p_*, where *A_p_ = 24.5 h_c_*^2^ is the projected area for a Berkovich-type indenter, and *F_max_* is the maximal applied load. To estimate the physical constants controlling phase-formation processes, the contact depth as *h_c_* at maximum load was considered to calculate both the hardness and the D [m^2^/s] parameter (see Table 4 and Table 5), recognizing that such loads do not exceed the depth of the coatings.

**Figure 13:**
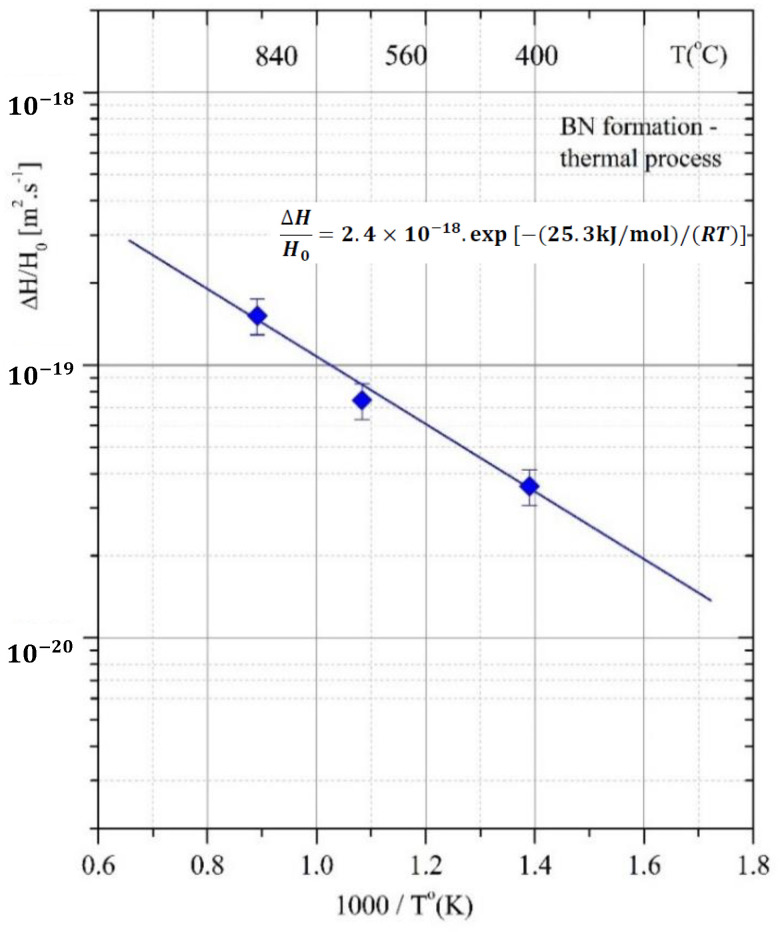
Arrhenius plot of BN phase formation for the annealed samples (1 h) in N_2_-atmosphere.

**Figure 14:**
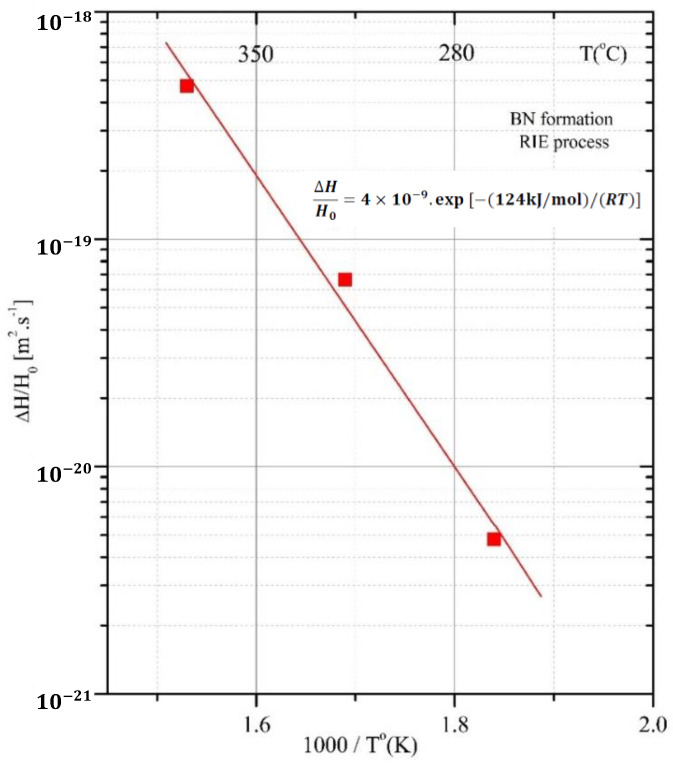
Arrhenius plot of BN phase-formation process via RIE plasma treatment for 10 min in an Ar−N_2_ mixture.

To describe the formation kinetics, Arrhenius plots were made for both thermal-annealing processes (Figure 13) and ion-milling processes (RIE) (Figure 14). The diffusion phenomenon is similar to that used to describe the rates of chemical reactions. It uses mathematical models and accurately describes physical phenomena.

A chemical reaction occurs, surmounting an energy barrier, where the thermodynamic probability, *P*, and the kinetic energy of the particles are described by Boltzmann-type formulas, such as *P* = *exp* (−ΔG*/RT). The free-energy difference, ΔG*(per mol), between normal and activated states is known as the activation free energy. Smaller ΔG* values and higher temperatures increase P exponentially and thus strongly enhance the prospects for atomic motion [54]. A proportional form of *P* has the diffusion coefficient introduced earlier, namely:

(1) $$D=D_{o}exp\left(-E_{A}/RT\right)$$

where *D_o_* is the temperature-independent pre-exponential factor and depends on how often (frequency) molecules collide when all concentrations are 1 mol/L and whether the molecules are properly oriented when they collide; and *E_A_* is the activation energy of the process and is used to describe the energy required to reach a transition state, also called “migration energy”. A higher value of *E_A_* means a “solid” or better barrier; or slower chemical reaction, including phase transformations. In the case of two adjacent media, a region *x* ≥ 0 of one substance and a region *x* < 0 of another substance exist, and the reaction/diffusion-penetration profile should conform to the solution of the diffusion equation for a pair of semi-infinite solids:

(2) $$H(x,t)=(H_{o}/2)erfc\left(x/[2{\left(Dt\right)}^{1/2}]\right)$$

(3) $$H(x,t)=(H_{o}/2)(1+erf\left(x/[2{\left(Dt\right)}^{1/2}]\right)$$

respectively, where *H(x,t)* is the hardness (*H*) at a depth, *x*, after the diffusion interval, *t*; *H_o_* is the initial value of the element/compound; and *D* its diffusivity; or Δ*H/H_o_* is the phase-formation process through its actual hardness value.

(4) $$erf\left(x\right)=\frac{2}{\sqrt{\pi }}\int_{0}^{x}e^{-t^{2}}dt$$

Equation (4) refers to the error function, while *erfc(x) =* 1 − *erf(x)* is the complementary error function. The rewritten form of (2)—*erfc* ^−1^(2*H/H_o_) = x*/[2*(Dt)*^1/2^]—is a linear function of the depth, *x*, and allows for the assessment of *D* (see Table 4 and Table 5). Hence, the process rates of BN phase formation evaluated considering the film-hardness dynamic for both annealed and RIE-treated samples are:

(5) $$\frac{\Delta H}{H_{o}}=2.4\times 10^{-18}exp[-\left(25.3\;\mathrm{kJ}/\mathrm{mol}\right)/\left(RT\right)]$$

(6) $$\frac{\Delta H}{H_{o}}=4\times 10^{-9}exp[-\left(124\;\mathrm{kJ}/\mathrm{mol}\right)/\left(RT\right)]$$

respectively, where *R*, the gas constant = 8.314 × 10^−3^ [kJ/mol °K], which is similar to the Boltzmann constant, *k_B_*, which relates the average relative kinetic energy of particles in gas/solids with its thermodynamic temperature. Figure 13 presents relation Equation (5) and refers to the fitted data shown in Table 4. The pre-exponential coefficient (2.4 × 10^−18^) [m^2^/s] refers to the rate of phase formation, similar to diffusion rate. Grigorov et al. [55] reported an analogous process rate in the temperature range of 400–900 °C for silicon diffusion in TiN films. *D_o_* is called the frequency factor or the attempt frequency of the reaction and accounts for the total number of collisions (leading to a reaction or not) per second. Therefore, the denser the barrier/substrate (free of defects), the fewer collisions with inner atoms. In the same report, the authors alleged that TiN thin films with different microstructures show different diffusivity coefficients. The B+ and Bo TiN films deposited with and without the ion-assisted PVD process show diffusivity from 400 to 900 °C, respectively:

$$D\left(m^{2}s^{-1}\right)=2.5\times 10^{-18}exp[-\left(31\;\mathrm{kJ}/\mathrm{mol}\right)/\left(RT\right)]$$

and

$$D\left(m^{2}s^{-1}\right)=3\times 10^{-19}exp[-\left(26\;\mathrm{kJ}/\mathrm{mol}\right)/\left(RT\right)]$$

The obtained activation energies are typical for grain-boundary diffusion [53]. According to Sarah Khalil et al., Ag atoms appeared to follow the Σ3 grain-boundary transport process in SiC substrate annealed up to 1300 °C, and the diffusivity of 4 × 10^−20^ (m^2^/s) is a result of a process partially controlled by grain-boundary diffusion [56].

According to Table 5, the RIE process occurred at lower temperatures. The N_2_-induced RF plasma should play an important role in the transformation of the B-containing coating into BN film. Moreover, the samples were subjected to a negative bias from 200 to 400 eV. The greater difference in the activation energy for the RIE (124 kJ/mol vs. 25.3 kJ/mol) suggests pronounced surface diffusion in the voids arising during this process. A similar *E_A_* value was obtained for the BN formation via CVD (115.1 kJ/mol), and the process was controlled by the surface chemical-reaction kinetics [57]. The RIE process occurred with relatively denser plasma (Ar + N_2_) of 13.3 Pa (100 mTorr), promoting a shallow zone of microcracks. The changes in the near-surface morphology of RIE-treated samples evokes, for this temperature region, a non-typical temperature-dependent process (see the slope of the fitted curve in Figure 14), which does not ensure enough time (and sufficient temperature) for the desired phase formation. Moreover, ion bombardment could result in resputtering of the constituent species, where both consist of light elements.

To relate the obtained results and gain a better understanding of the impact of both the thermal and ion treatment, a multiple linear regression was considered using two independent variables of temperature: polarization during RIE and hardness. The equation from the type is *z* = *a*_0_ + *a*_1_*x* + *a*_2_*y*, where *z* is associated with the hardness (*H*), and *a*_0_ = *H_o_* yields the coefficients of the independent variables: *a*_0_ = 5.14; *a*_1_ = 0.52; *a*_2_ = 0.19; or H=5.14+0.52T+0.19P. Table 6 summarizes these results. Apparently, the temperature factor appears to be almost three times more efficient than ion bombardment for c-BN phase formation. For the independent tribological data (FC), a probable reassessment needs to be undertaken or ion bombardment, in an indirect manner, provides conditions for lowering the friction coefficient. It appears that both techniques should act synergistically. A technique that supplies simultaneous action of both processes could be reactive magnetron sputtering (e.g., HiPIMS) at elevated temperatures.

## 4. Conclusions

Nanocrystalline h-BN and c-BN thin films were obtained on polished AISI 1045 carbon-steel samples covered with B-containing thin organic film transformed by means of two methods: thermal annealing at 450–850 °C and reactive ion etching (RIE) in Ar/N_2_ plasma. Detailed analyses of coating morphology (SEM), crystalline structure (XRD), chemical composition (XPS, EDS), and tribology (FC) were conducted. Post-annealing at 450 °C did not lead to the formation of a BN phase in the layer. A non-stoichiometric BN phase with a nitrogen deficiency appeared at 650 °C. At 850 °C annealing, the formed BN phase was completely stoichiometric. Hardness, BN phase-formation rate (D_o_), and activation energy (E_A_) were studied. The RIE-treated samples exhibited a few orders greater growth dynamic rate 4 × 10^−9^ (m^2^ s^−1^) than the temperature-treated samples 2.4 × 10^−18^ (m^2^ s^−1^), as well as greater energy of activation (124 kJ/mol vs. 25.3 kJ/mol) associated with pronounced surface diffusion in the voids developed during the RIE process. The temperature-treated samples showed markedly higher hardness (up to 10 GPa) and higher friction coefficients than the RIE-treated (0.65 vs. 0.45). To relate the obtained results to each other and gain a better understanding of the impact of the thermal and ionic treatment processes, a multiple linear regression was performed with two independent variables of the temperature, the polarization during the RIE and the hardness values were considered. The temperature factor appears to be almost three times more efficient than ion bombardment for c-BN phase formations, but the latter lowers the friction coefficient in an indirect manner.

## Figures and Tables

**Table 1:** EDS semiquantitative elemental composition of the selected particles in Figure 5c and Figure 6c correspond to Sample 2 (650 °C) and Sample 3 (850 °C), respectively.

Point/Sample	B	O	Na	Mg	Si	K	Fe
2/2	0	33.17	0	1.9	0	0	64.93
3/2	38.67	37.39	1.02	1.87	0.41	0.2	20.43
4/2	46.92	33.1	0.77	1.57	0.38	0.16	17.12
7/3	33.67	45.78	0.55	1.18	0.54	0.11	18.18
8/3	0	48.82	0	1.61	0	0	49.56
9/3	36.27	42.7	0.93	1.41	1	0.06	17.62

**Table 2:** Detailed elemental concentration deduced from XPS. Deconvolution of signals from each film constituent used.

Sample	C (at. %)	O (at. %)	N (at. %)	B (at. %)	Fe (at. %)	Na (at. %)
1/450 °C	52.6	32.5	1.8	9.1	4.2	0.0
2/650 °C	23.4	45.5	4.6	24.1	1.1	1.1
3/850 °C	30.1	39.2	5.2	23.3	1.0	1.2

**Table 3:** Percentage of boron phases in each sample.

Sample	Peak Position [BE]	Integrated Area [a.u]	Part of the Whole Spectrum in %	Type of Phase
1–450 °C	186.6	124.1	24.8	BC
	188.4	125.5	25.1	Fe_2_B, FeB
	191.1	250.1	50.1	Organic B
2–650 °C	186.0	180.6	2.9	B_4_C
	188.4	629.1	10.1	Fe_2_B, FeB
	190.0	1905.5	30.6	BN
	191.2	2376.8	38.2	Organic B
	192.6	1135.5	18.2	Na_2_B_4_O_7_ · 10H_2_O
3–850 °C	186.0	6.37	6.4	B_4_C
	188.9	18.84	18.8	Fe_2_B, FeB
	190.3	17.95	18.0	BN
	191.1	49.63	49.6	Organic B
	192.5	7.20	7.2	Na_2_B_4_O_7_ · 10H_2_O

**Table 4:** Growth rate of the BN phase for the annealed samples at different temperatures in running N_2_. Referent value of H_o_ = 5.39 GPa.

Sample	Temp [°C]	Depth [nm]	ΔH/H_o_	erfc^−1^	D [m^2^s^−1^]
A1	450	22.8	0.437	0.509	3.6 × 10^−20^
A2	650	32.8	0.579	0.392	7.44 × 10^−20^
A3	850	46.8	0.729	0.243	1.52 × 10^−19^

**Table 5:** Growth rate of the BN phase for the plasma-treated samples in an Ar/N_2_ mixture for 600 sec at different RF powers. Referent value of H_o_ = 5.39 GPa.

Sample	Power [W]	U_bias_ [V]	Temp [°C]	Depth [nm]	ΔH/H_o_	erfc^−1^	D [m^2^s^−1^]
A100	100	210	270	3.4	−0.19	5.89	4.8 × 10^−21^
A200	200	310	320	12.6	−0.044	2.09	6.65 × 10^−20^
A300	300	380	380	33.6	0.11	1.13	4.7 × 10^−19^

**Table 6:** Multiple linear regression of two independent variables.

**T °C/100**	x	0	4.5	6.5	8.5	0	0	0
**Pol (eV)/100**	y	0	0	0	0	2.1	3.1	4.5
**H(GPa)**	z	5.39	7.94	8.51	9.32	4.62	5.79	6.35

## Data Availability

The data that support the findings of this study are available from the corresponding author on reasonable request.
